# Emerin Caps the Pointed End of Actin Filaments: Evidence for an Actin Cortical Network at the Nuclear Inner Membrane

**DOI:** 10.1371/journal.pbio.0020231

**Published:** 2004-08-24

**Authors:** James M Holaska, Amy K Kowalski, Katherine L Wilson

**Affiliations:** **1**Department of Cell Biology, The Johns Hopkins University School of MedicineBaltimore, MarylandUnited States of America

## Abstract

X-linked Emery-Dreifuss muscular dystrophy is caused by loss of emerin, a LEM-domain protein of the nuclear inner membrane. To better understand emerin function, we used affinity chromatography to purify emerin-binding proteins from nuclear extracts of HeLa cells. Complexes that included actin, αII-spectrin and additional proteins, bound specifically to emerin. Actin polymerization assays in the presence or absence of gelsolin or capping protein showed that emerin binds and stabilizes the pointed end of actin filaments, increasing the actin polymerization rate 4- to 12-fold. We propose that emerin contributes to the formation of an actin-based cortical network at the nuclear inner membrane, conceptually analogous to the actin cortical network at the plasma membrane. Thus, in addition to disrupting transcription factors that bind emerin, loss of emerin may destabilize nuclear envelope architecture by weakening a nuclear actin network.

## Abstract 2

Loss of emerin leads to Emery-Dreifuss muscular dystrophy (EDMD). Biochemical studies presented here suggest that emerin drives the formation of an actin-based cortical network at the nuclear membrane, and that network destabilization may contribute to EDMD

## Introduction

Emery-Dreifuss muscular dystrophy (EDMD) is inherited through mutations in either of two different genes: *LMNA,* encoding A-type lamins, and *STA,* which encodes a nuclear membrane protein named emerin (Nagano et al. 1996; Emery 2000; Bengtsson and Wilson 2004). Lamin filaments and emerin interact at the nuclear inner membrane (Burke and Stewart 2002; Holaska et al. 2002). Together, emerin and lamin A form stable tertiary complexes with other binding partners in vitro (Holaska et al. 2003), suggesting that emerin and lamins together provide a structural foundation for oligomeric protein complexes. Mutations in emerin cause the X-linked recessive form of EDMD (Bione et al. 1995; Bonne et al. 2003). Both emerin and lamin A are expressed in most cells, but EDMD disease strikes specific tissues: skeletal muscles, major tendons, and the cardiac conduction system. To explain the tissue specificity of EDMD, it was proposed that emerin might have tissue-specific binding partners such as transcription factors and signaling molecules that regulate gene expression (Wilson 2000; Bonne et al. 2003; Östlund and Worman 2003). There is growing evidence to support “gene expression” models for emerin, as discussed further below. However, a second model, not mutually exclusive, proposes that emerin helps maintain the structural integrity of the nuclear envelope. According to structural models, loss of emerin selectively disrupts tissues under high mechanical stress, such as skeletal muscle and tendons (Bonne et al. 2003; Östlund and Worman 2003). Although this model fails to explain the cardiac conduction phenotype of EDMD, it is consistent with structural defects (aberrant shape and nuclear envelope herniations) seen in nuclei from EDMD patients (Fidzianska and Hausmanowa-Petrusewicz 2003) and in a subset of patients with other diseases linked to mutations in *LMNA* (“laminopathies”; Holaska et al. 2002; Östlund and Worman 2003). Whereas structural and mechanical roles are expected for lamins, which form nuclear intermediate filaments, mechanical roles for emerin have not been investigated.

Emerin is detected in most human cells tested, except the nonmyocytes of the heart (Manilal et al. 1996). In Caenorhabditis elegans emerin is expressed ubiquitously (Lee et al. 2000; Gruenbaum et al. 2002). Emerin belongs to the LEM-domain family of proteins, which are defined by an approximately 40-residue folded domain known as the LEM domain. In vertebrates, other family members include LAP2β and MAN1 at the nuclear inner membrane (Dechat et al. 2000; Lin et al. 2000; Cohen et al. 2001), LAP2α in the nuclear interior (Dechat et al. 2000), and at least three additional uncharacterized LEM-domain proteins (Lee and Wilson 2004). A major shared function of all characterized LEM-domain proteins is their binding (via the LEM domain) to a small protein named barrier-to-autointegration factor (BAF; reviewed by Segura-Totten and Wilson 2004). BAF is a highly conserved chromatin protein essential for cell viability (Zheng et al. 2000), with direct roles in higher order chromatin structure and nuclear assembly (Haraguchi et al. 2001; Segura-Totten et al. 2002), and gene regulation (Wang et al. 2002; Holaska et al. 2003).

Supporting gene regulation models for EDMD, emerin binds directly to BAF and two other transcription repressors, germ cell-less (GCL; Holaska et al. 2003) and Btf (Haraguchi et al. 2004) as well as an mRNA-splicing factor named YT521-B (Wilkinson et al. 2003). Interestingly, GCL and LAP2β, a LEM-domain protein closely related to emerin, comediate transcription repression in vivo (Nili et al. 2001). On the other hand, emerin also has a growing number of structural or anchoring partners, including a spectrin-repeat (SR) membrane protein named nesprin-1α (Mislow et al. 2002a, 2002b), lamins A and C (Clements et al. 2000; Lee et al. 2001; Sakaki et al. 2001), and lamin B (Dreger et al. 2002). Lamins form type-V intermediate filaments that are critical for the integrity of the nucleus and confer unique elasticity and incompressibility properties to the nuclear envelope (Dahl et al. 2004). Nuclear morphology and lamina architecture are disrupted in a fraction of cells that express disease-causing missense mutations in A-type lamins (Östlund and Worman 2003). However, the line between gene expression and mechanical models for disease has become blurred. For example, the subnuclear localization of Rb, a transcription and cell-fate regulator, depends on both LAP2α and lamin A, since Rb is mislocalized in cells that either lack A-type lamins (Johnson et al. 2004) or express disease-causing lamin-A mutations (Markiewicz et al. 2002). A second putative anchoring partner for emerin is nesprin-1α, an integral nuclear inner-membrane protein with seven SR domains (Zhang et al. 2001; Mislow et al. 2002b). Each SR domain consists of approximately 100 residues and folds into a tightly packed triple α-helical structure (Bennett and Baines 2001). Tandem SR domains, as seen in nesprin-1α, form a rigid, elongated tertiary structure (Djinovic-Carugo et al. 2002). More important, SR domains provide binding sites for other proteins, the specificity of which is determined by exposed residues (Bennett and Baines 2001). SR domains 1–7 (and particularly domains 1–5) of nesprin-1α mediate high-affinity binding to emerin (Mislow et al. 2002a). Interestingly, SR domains 5–7 of nesprin-1α bind directly to lamin A in vitro, suggesting that nesprin-1α, lamin A, and emerin might form stable tertiary complexes. Such complexes have the potential to stabilize lamin filaments at the nuclear envelope, in addition to anchoring and spacing emerin.

Notably, both emerin and lamin A also bind G-actin in vitro (Sasseville and Langelier 1998; Fairley et al. 1999). Actin binds two regions in the lamin-A tail (Zastrow et al. 2004). Both α- and β-actin bind emerin in vitro, and emerin coimmunoprecipitates with actin from cell lysates (Fairley et al. 1999; Lattanzi et al. 2003). The significance of these findings was unclear, in part because nuclear actin has been both documented and debated for over 35 y (Pederson and Aebi 2002). However there is a growing consensus that nuclear actin is no artifact (Pederson and Aebi 2002; Bettinger et al. 2004). Both α- and β-actin have been shown, definitively, to reside in the nucleus (Scheer et al. 1984; Gonsior et al. 1999; Olave et al. 2002; Lattanzi et al. 2003) and to form short filaments in the nucleus (Clark and Rosenbaum 1979). Actin and actin-related proteins (Arps) are required for chromatin remodeling and transcription (Olave et al. 2002; Percipalle et al. 2003). Also interesting is that polymerase II–dependent mRNA transcription requires a nuclear-specific myosin I motor (nuclear myosin I; Nowak et al. 1997; Pestic-Dragovich et al. 2000). Thus, actin probably has a variety of roles in the nucleus.

To test the hypothesis that emerin forms multiprotein complexes in vivo, we affinity-purified emerin-binding proteins from nuclear extracts of HeLa cells. We identified actin itself plus several actin-binding proteins as bona fide emerin-associated proteins, and we further discovered that emerin stimulates actin polymerization in vitro by binding and stabilizing the pointed end of growing filaments. These results suggest that emerin contributes to the formation of an actin cortical network at the nuclear inner membrane.

## Results

**Figure 1:**
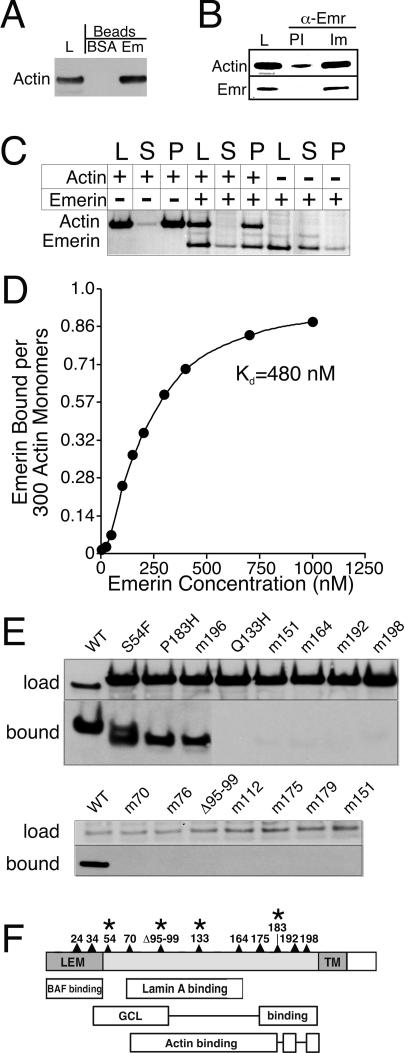
Affinity Purification of Emerin-Associated Proteins (A) Immunoblot of HeLa nuclear lysate proteins (L), or proteins affinity-purified using either BSA beads or emerin beads (see Materials and Methods), probed with antibody against actin. (B) HeLa nuclear lysate proteins (L) were immunoprecipitated using either immune (Im) or preimmune (PI) serum 2999 against emerin, resolved by SDS-PAGE, and Western blotted using antibodies specific for actin (upper panel) or emerin (lower panel), in succession. (C) Cosedimentation assays using F-actin and purified, recombinant wild-type emerin (residues 1–222). G-actin (2 μM) was polymerized and then incubated in the absence or presence of 2 μM emerin. Emerin was incubated alone in polymerization buffer as a negative control. After 30 min samples were centrifuged 1 h at 100,000*g,* resolved by SDS-PAGE, and stained with Coomassie blue. L, load (100%); S, supernatant (100%); P, pellet (100%). (D) F-actin column was used to determine the affinity of F-actin for emerin. The K_d_ was 480 nM for the experiment shown; range was 300–500 nM, *n* = 8. (E) Binding of wild-type (WT) or mutant emerin protein to F-actin beads. Recombinant emerin proteins were incubated with F-actin beads, and bound emerins were eluted with SDS-PAGE sample buffer, resolved by SDS-PAGE, blotted, and probed with antibodies against emerin (“bound”; all emerin mutants are recognized by this antibody; Lee et al. 2001; Holaska et al. 2003). The input amounts (10%) of each emerin mutant (“load”) were visualized either by immunoblotting (top row, top panel) or Coomassie staining (top row, bottom panel). (F) Domains in emerin required for binding to BAF, lamin A, transcription repressor GCL, or actin (Lee et al. 2001; Holaska et al. 2003; present study). Asterisks indicate EDMD disease-causing mutations.

We used affinity chromatography to purify emerin-binding complexes from HeLa nuclear extract; as the negative control, beads were conjugated to bovine serum albumin (BSA). Mass spectrometry (data not shown) and Western blotting identified β-actin as a major emerin-binding protein (Figure 1A). Six other proteins, including nuclear-enriched αII-spectrin, were also identified and will be reported in full elsewhere (J. M. H. and K. L. W., unpublished data). Consistent with previous reports (Fairley et al. 1999; Lattanzi et al. 2003), antibodies specific for emerin coprecipitated actin from HeLa cell nuclear lysates, as shown by Western blotting with immune serum (Figure 1B, Im). Only background levels of actin were precipitated by preimmune sera (Figure 1B, PI). These findings led us to hypothesize that emerin might bind filamentous actin (F-actin).

### Emerin Binds F-Actin with High Affinity

Emerin was first tested for binding to F-actin in a cosedimentation assay. Actin filaments were incubated 30 min in the presence or absence of recombinant emerin (4 μM) and then pelleted at 100,000*g.* Approximately 75% of input emerin pelleted in the presence of F-actin, compared to 15% in the absence of F-actin (Figure 1C), demonstrating that emerin binds polymerized actin in vitro. The stoichiometry of this interaction was one emerin molecule per approximately 300 actin monomers, demonstrating that emerin binds actin filaments with an average length of 0.9 μm (data not shown). Emerin binds F-actin with high affinity, as determined by binding to an F-actin column (K_d_ = 480 nM, range = 300–500 nM, *n* = 8; Figure 1D). The F-actin columns were also used to screen selected emerin mutants for binding to F-actin. Wild-type emerin and EDMD disease-causing mutants S54F and P183H, and alanine substitution mutant m196 (Ser^196^Ser^197^ to Ala^196^Ala^197^; Holaska et al. 2003), bound efficiently to an F-actin column, whereas 12 other tested mutants, including EDMD disease-causing mutant Q133H, showed no significant binding to F-actin (Figure 1E). Similar results were seen in coimmunoprecipitation assays using antibodies against actin to immunoprecipitate F-actin in the presence of recombinant wild-type or mutant emerin proteins (data not shown). Fifteen additional emerin missense mutants, including LEM-domain mutants, were tested in blot overlay assays; mutants m11, m24, m30, m40, m207, m214, and m217 bound detectably to actin, whereas no significant binding was detected for mutants m45A, m45E, m61, m141, m145, m161, and m206 (data not shown; see Holaska et al. 2003 for details of mutations). Thus, three independent assays all showed that emerin binds F-actin. Furthermore, based on the positions of missense mutations that blocked binding to F-actin, this recognition involved almost the entire nucleoplasmic domain of emerin, with the notable exception of the LEM-domain (Figure 1F). The putative actin-binding domain in emerin overlaps with both the lamin- and repressor-binding (GCL) domains described previously (Holaska et al. 2003).

### Emerin Regulates Actin Polymerization

**Figure 2:**
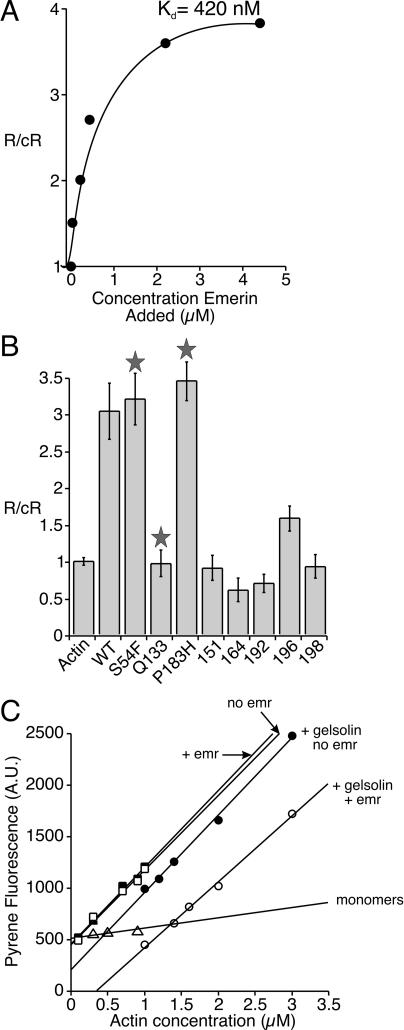
Emerin Stimulates Actin Polymerization (A) Graph of a representative experiment (*n* = 32) showing that emerin increases the rate of actin polymerization 4-fold. R, rate of polymerization in the presence of emerin; cR, control rate of polymerization (actin alone). These data also yielded an equilibrium binding affinity of emerin for actin of 420 nM. (B) Representative graph (*n* = 17) in which each recombinant emerin mutant protein (1.0 μM) was added to 2.0 μM G-actin, and polymerization rates were calculated. R, rate of polymerization in the presence of emerin; cR, control rate of polymerization (actin alone). Stars indicate EDMD disease-causing mutations. (C) Critical concentration assays were performed in the presence or absence of 625 nM emerin. Actin was polymerized in the absence (barbed-end growth) or presence of 5 nM gelsolin–actin seeds (pointed-end growth) for 16 h at room temperature. Barbed-end growth with (□) or without (▪) emerin. Pointed-end growth with (○) or without (•) emerin. Actin monomers (Δ).

The above results suggested that in vivo emerin might (a) use actin filaments as anchors, (b) stabilize F-actin networks, or (c) actively influence actin dynamics. To test these models, we first used reactions containing 5% pyrene-labeled actin (final actin concentration, 2 μM) to determine if emerin influenced actin polymerization in vitro. Results were graphed as the rate of emerin-induced polymerization (R) divided by the control rate (cR; rate of actin polymerization in the absence of emerin). At concentrations ranging from 0.1 to 4.4 μM, emerin increased the rate of pyrene–actin polymerization 4-fold (mean = 6.2 ± 2.2, *n* = 32; one experiment is shown in Figure 2A). These experiments also yielded an equilibrium affinity of 420 nM (Figure 2A), consistent with our previous results (480 nM; see Figure 1D).

We next tested eight emerin mutants (1 μM) in the pyrene–actin polymerization assay (Figure 2B). Five mutants (Q133H, m151, m164, m192, and m198) that failed to bind actin in vitro did not stimulate actin polymerization; instead, they reduced the rate of actin polymerization slightly, by 5%–40% (Figure 2B). Mutant 196, which had wild-type binding to F- and G-actin in coimmunoprecipitation assays, stimulated actin polymerization approximately 50% as well as wild-type emerin (Figure 2B). The two disease-causing mutants with apparently normal binding to F-actin, S54F and P183H, enhanced the rate of actin polymerization at least as well as wild-type emerin (Figure 2B). Critical concentration assays were done to determine if emerin acted on the barbed or pointed end of growing actin filaments. Pointed-end growth was examined by capping filaments with gelsolin. Emerin had no significant effect on the critical concentration of barbed-end growth (Figure 2C, + emr/no emr), but it increased the critical concentration for pointed-end growth by 2.3- to 2.7-fold (Figure 2C, + gelsolin no emr/+ gelsolin + emr). We therefore hypothesized that emerin, like tropomodulin (Fowler et al. 2003), might stabilize growing filaments by capping the pointed end.

### Emerin Binds F-Actin at the Pointed End

**Figure 3:**
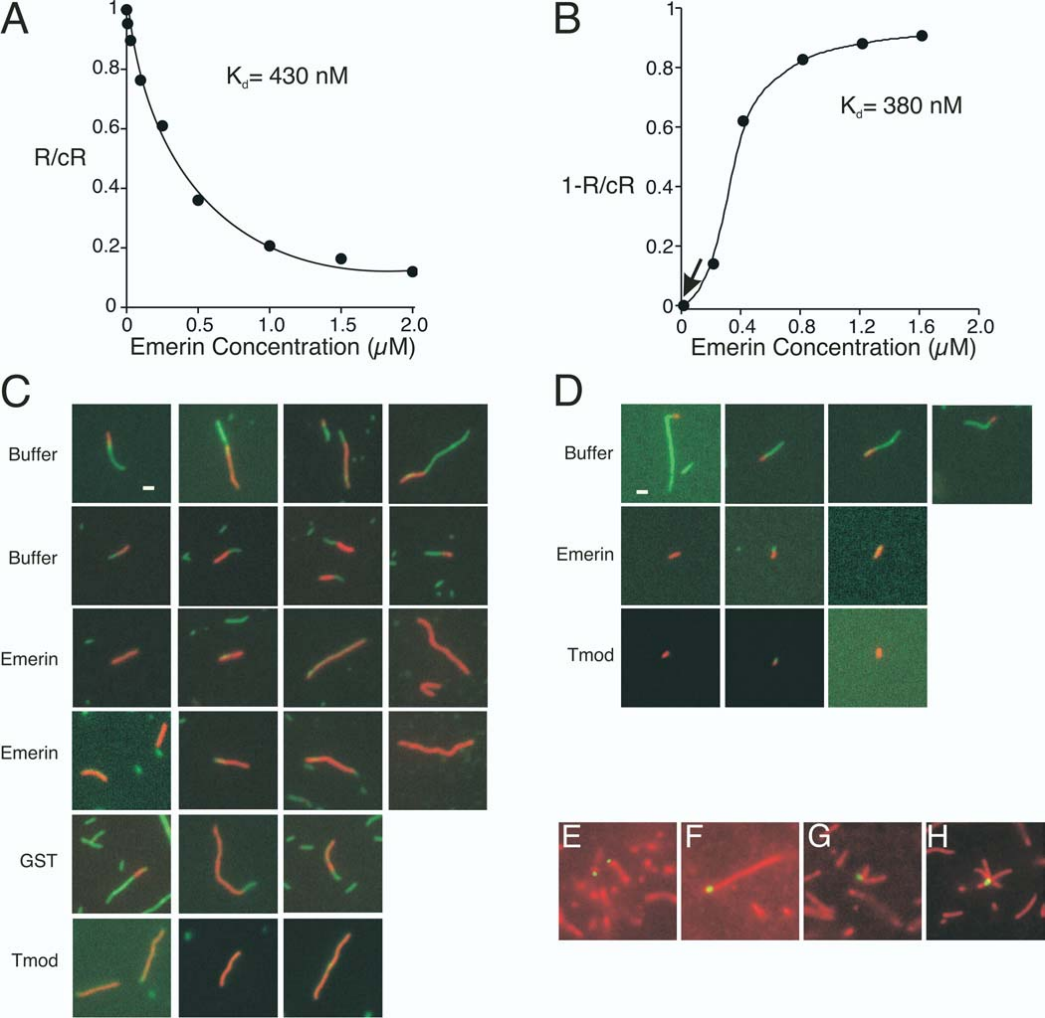
Emerin Binds the Pointed End of Actin Filaments (A) Gelsolin–actin seeds were incubated with increasing concentrations of wild-type emerin residues 1–222. Emerin significantly reduced the rate of subunit addition at the pointed end, with an apparent K_d_ of 430 nM (range, 300–500 nM, *n* = 12). R, rate of polymerization in the presence of emerin; cR, control rate of polymerization (actin alone). (B) Emerin inhibits depolymerization of actin filaments (2 μM) preformed from gelsolin–actin seeds, with an apparent K_d_ of 380 nM (range, 350–450 nM, *n* = 6). R, rate of depolymerization in the presence of emerin; cR, control rate of depolymerization (actin alone). (C) Rhodamine–phalloidin-stabilized actin filaments were formed from 2 μM actin, then capped at the barbed end by the addition of 100 nM capping protein, and finally diluted 2-fold in the presence of buffer or 1 μM emerin, GST, or tropomodulin (Tmod). Samples were then incubated with actin (3.2 μM) and Alexa-488 phalloidin (3.2 μM) for 2 min, diluted 1:500, placed on polylysine-coated coverslips, and viewed by fluorescence microscopy. Bar is 1 μm and applies to all panels. (D) Actin (2 μM) was incubated with gelsolin–actin seeds (500 nM) in the presence of rhodamine–phalloidin (2 μM). These red filaments were diluted 10-fold and incubated with buffer alone or with 1 μM emerin or tropomodulin (Tmod) for 10 min, followed by incubation with actin (2 μM) and Alexa-488-labeled (green) phalloidin (2 μM) for 2 min. Samples were diluted 1:500, placed on polylysine-coated coverslips, and viewed by fluorescence microscopy. Bar is 1 μm and applies to all panels. (E–H) Alexa-488-labeled emerin (green) was incubated 30 min with actin filaments stabilized by Alexa-546 phalloidin (red) and centrifuged at 100,000*g* to recover filaments, which were diluted 1:500 for viewing.

The vast majority of actin-binding proteins that influence subunit addition do so by binding the barbed end (dos Remedios et al. 2003). However, because emerin failed to influence the critical concentration for barbed-end growth (Figure 2C), we used gelsolin–actin seeds to test the hypothesis that emerin binds the pointed end. Gelsolin binds and caps the barbed end of actin filaments (Burtnick et al. 2001), thereby restricting subunit addition to the pointed end only. We measured the extension of gelsolin–actin dimers (10 nM) in the presence of 2 μM actin plus 0 to 2 μM wild-type emerin (Figure 3A). Emerin blocked actin polymerization in a concentration-dependent manner, supporting our model that emerin binds and caps the pointed end of actin filaments. Based on this assay, the affinity (K_d_) of emerin for F-actin was 430 nM (range, 300–500 nM, *n* = 12; Figure 3A). Interestingly, the affinity determined by either direct binding (see Figure 1D) or activity measurements (see Figures 2A and 3A) differed by a maximum of 2-fold. We conclude that emerin binds F-actin with an affinity of 300–500 nM.

To independently confirm that emerin binds the pointed end, we tested the effect of emerin on actin depolymerization in two separate assays. First, gelsolin–actin seeds were incubated with 2 μM actin and grown in the absence of emerin. The resulting filaments were then diluted to 0.2 μM actin in the presence of increasing concentrations of emerin (0–2 μM), and assayed immediately. Preformed actin filaments depolymerized rapidly in the absence of emerin (Figure 3B, arrow), as expected. Supporting our model, depolymerization was slowed up to 8-fold by emerin (Figure 3B). To independently confirm that emerin blocked subunit addition at the pointed end, preformed actin filaments were capped at the barbed end with capping protein (CapZ), a high-affinity barbed-end binding protein (Cooper and Schafer 2000), then incubated in the presence or absence of emerin, and diluted into 0.2 μM actin; emerin slowed the rate of depolymerization by 10-fold (data not shown). Based on these three assays, we conclude that emerin binds and protects the pointed end of actin filaments in vitro, thereby stabilizing actin filaments.

Fluorescent actin polymerization assays were done to demonstrate visually that emerin blocks pointed-end growth of single actin filaments. Rhodamine–phalloidin-stabilized actin filaments (red) were preformed from 2 μM actin, then capped on the barbed end with capping protein, and diluted 2-fold into a final concentration of 1 μM emerin. Pointed-end growth was then initiated by increasing actin to 3.2 μM in the presence of 3.2 μM Alexa-488 phalloidin (green) for 2 min. In the absence of emerin (Figure 3C, buffer or GST), actin filaments containing both red and green segments are seen (Figure 3C), demonstrating pointed-end growth. The average lengths of the growing filament segments (green) as measured for buffer and GST were 1.45 ± 0.3 μm and 1.47 ± 0.3 μm, respectively (*n* = 30). However, in the presence of either emerin or tropomodulin, a pointed-end binding protein, most red filaments lacked green segments (Figure 3C), consistent with capped pointed ends. In the presence of emerin or tropomodulin, the lengths of the green segments were 0.05 ± 0.09 μm (*n* = 60) and 0.09 ± 0.13 μm (*n* = 50), respectively. Single filament assays were also done using small red filaments formed from gelsolin–actin seeds (Figure 3D). Here, actin (2 μM) was incubated with gelsolin–actin seeds (500 nM) and rhodamine–phalloidin (2 μM). The resulting red filaments were diluted 10-fold and incubated with 1 μM emerin for 10 min, then incubated 2 min with actin (2 μM) and Alexa-488-labeled (green) phalloidin (2 μM). In the absence of emerin, single filaments contained both short red (gelsolin–actin seeds) and longer green (pointed-end growth) segments (Figure 3D). The average length of these growing filament segments was 2.1 ± 0.6 μm (*n* = 40). However, when emerin was present, the preformed filaments remained predominantly short and red, demonstrating that emerin blocks pointed-end growth (Figure 3D). Similar results were obtained in control reactions containing tropomodulin, the pointed-end binding protein (Figure 3D, Tmod). The average lengths of growing filaments incubated with emerin or tropomodulin were 0.1 ± 0.1 μm (*n* = 40) and 0.1 ± 0.11 μm (*n* = 40), respectively. These experiments also show that emerin does not stimulate branching (Figure 3D). To directly visualize emerin bound to actin filaments, green (Alexa-488-labeled) emerin was incubated with red (Alexa-546 phalloidin) actin filaments (Figure 3E–3H). Under these conditions 85% of labeled emerin molecules were bound to actin filaments; of these, 92% were localized at a filament end (*n* = 306). Interestingly, 10% of actin-associated emerin proteins localized to “aster-like” structures (Figure 3G and 3H), presumably due to the aggregation of emerin proteins on different actin filaments.

## Discussion

This work shows for the first time that a nuclear membrane protein, emerin, is a pointed-end F-actin-binding protein. Similar to the activity of tropomodulin (Fowler 1997; Cooper and Schafer 2000; Fowler et al. 2003), emerin caps the pointed end, thereby stabilizing the growing filament. Only three other pointed-end binding proteins have been reported: the Arp2/3 complex (Mullins et al. 1998), tropomodulin, and mSWI/SNF, a component of a nuclear complex that remodels chromatin structure (Rando et al. 2002). The Arp2/3 complex initiates filament branching at the cell surface (Mullins et al. 1998; Mullins and Pollard 1999). We have no evidence that emerin initiates branching. Instead, emerin behaves most like tropomodulin, which binds the pointed end of F-actin with high affinity (K_d_ = 110 nM) and stimulates actin polymerization by stabilizing the actin filament (Fowler et al. 2003).

Our analysis of 15 emerin missense mutants suggested that the actin-binding region in emerin overlaps with regions required for binding to lamin A (Lee et al. 2001), transcription factors GCL and YT521-B (Holaska et al. 2003; Wilkinson et al. 2003), and nesprin-1α (J. M. H. and K. L. W., unpublished data). However this overlap does not necessarily imply that actin competes with these other proteins. Indeed, despite similar overlap, GCL and lamin A can form stable ternary complexes with emerin in vitro (Holaska et al. 2003). Further work is needed to determine if F-actin cobinds or competes with lamin A, nesprin-1α, or other emerin-binding proteins.

### A Proposed Actin Network at the Nuclear Envelope

**Figure 4:**
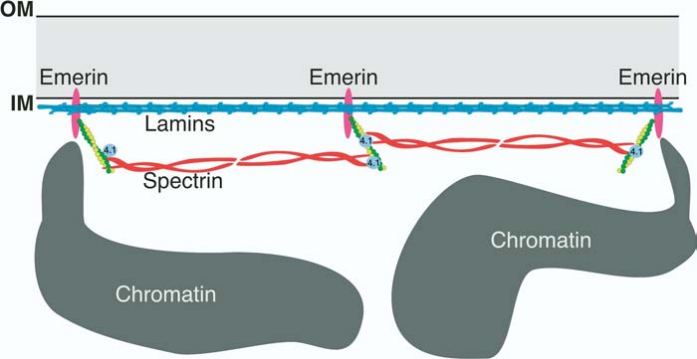
Model in Which Emerin Binding to the Pointed End of F-Actin Stabilizes an Actin Cortical Network at the Nuclear Inner Membrane Our model is based on the actin cortical network at the cell surface of erythrocytes, except that lamin filaments also anchor to emerin-based junctional complexes. Spectrin heterodimers bind short actin filaments at the erythrocyte membrane; we therefore speculate that nuclear isoforms of αII-spectrin (J. M. H. and K. L. W., unpublished data) act similarly. Direct binding of emerin and αII-spectrin has not yet been tested. Nuclear isoforms of protein 4.1, which are essential for nuclear assembly (Krauss et al. 2002), have the potential to cross-link short actin filaments and spectrin filaments at the inner membrane (IM). Further work is necessary to test our model and identify other components of this proposed nuclear actin cortical network. OM, nuclear outer membrane.

We propose that emerin stabilizes and promotes the formation of a nuclear actin cortical network, analogous to the actin cortical network at the plasma membrane (Figure 4). Another LEM-domain protein, LAP2β, also an integral nuclear inner-membrane protein, was 20-fold less active than emerin in actin polymerization assays (data not shown), suggesting that LAP2β binds actin with an affinity 20-fold lower than that of emerin. Other LEM-domain proteins have not yet been tested for binding to actin. Whether emerin has specialized roles involving actin, or shares this function with other nuclear membrane proteins, are both interesting possibilities. An actin-based cortical network could help anchor emerin and possibly other nuclear membrane proteins and lamin filaments, contributing significantly to the structural integrity of the nuclear envelope and potentially reinforcing sites of chromatin attachment (Figure 4).

Since lamin A also binds G-actin in vitro (Sasseville and Langelier 1998), we are currently testing the actin-binding properties of lamin A. Because emerin forms stable complexes with lamin A in vitro (Clements et al. 2000; Lee et al. 2001), and because the nuclear envelope localization of emerin depends on lamins, we speculate that emerin might interlink multiple filament networks (actin, spectrin, and lamins) at the nuclear envelope. This model will be tested in future experiments by determining whether lamin A and actin compete for binding to emerin, or form trimeric complexes. Such complexes could significantly reinforce the mechanical properties of the nuclear envelope. Our nuclear actin cortical network model is further supported by the properties of the nesprin family of nuclear membrane proteins, which includes nesprin-1α (see Introduction) and NUANCE. Nesprin-1α binds directly to both emerin (K_d_ = 4 nM) and lamin A (affinity undetermined; Mislow et al. 2002a). NUANCE is a large (796 kd), alternatively spliced isoform of nesprin that localizes to the nuclear envelope and nucleoplasm and binds F-actin (Zhen et al. 2002).

The organization of the membrane skeleton in erythrocytes (red blood cells) includes integral membrane proteins (e.g., Band 3), anchoring proteins (ankyrin), spectrin filaments, and “junctional complexes” (short actin filaments, protein 4.1, adducin, tropomodulin, and tropomyosin; Delaunay 2002). Tropomodulin and tropomyosin stabilize the junctional complex. Spectrin filaments (α/β-spectrin heterodimers) attach to junctional complexes through direct binding to protein 4.1, adducin, and actin. At the inner nuclear membrane, our working model is that emerin stabilizes junctional complexes (Figure 4), consisting of short actin filaments, nuclear-specific αII-spectrin (McMahon et al. 1999), and nuclear isoforms of protein 4.1 (Krauss et al. 2002). This model is the first step toward understanding the structural function of nuclear actin.

## Materials and Methods

### 

#### Antibodies and proteins

A pan-actin antibody (Sigma, St. Louis, Missouri, United States; catalog #A-5060) was used at 1:1,000 for immunoblotting. An antibody specific for β-actin (Sigma; catalog #A-5316) was used at 1:10,000 for immunoblotting and 1:1,000 for immunoprecipitation. Our rabbit polyclonal emerin antibody (serum 2999), described previously (Lee et al. 2001), was used at 1:20,000 for immunoblotting and 1:2,000 for immunoprecipitation. Purified chicken actin was a kind gift of Doug Robinson (Johns Hopkins Medical School). Purified rabbit actin was purchased from Cytoskeleton. (Denver, Colorado, United States; catalog #AKL95 and #AKL99). Alexa-488 actin (#A12373), Alexa-594 actin (#A34050), Alexa-488 phalloidin (#A-12379), rhodamine–phalloidin (#R-415), and Alexa-546 phalloidin (#A-22283) were purchased from Molecular Probes (Eugene, Oregon, United States). Purified CapZ (capping protein) was a kind gift from John Cooper (Washington University, St. Louis). The full nucleoplasmic domain of wild-type emerin (residues 1–222) and corresponding mutants (detailed in Holaska et al. 2003) were expressed in bacteria and purified as described (Lee et al. 2001; Holaska et al. 2003). Emerin protein was labeled with Alexa-488 (Molecular Probes, catalog #A20000) per manufacturer's instructions.

#### Affinity purification using emerin-conjugated beads

Wild-type emerin residues 1–222 (comprising the entire nucleoplasmic domain of emerin and lacking the transmembrane domain) or BSA (as a negative control) were coupled to Affigel-15 beads (Bio-Rad, Hercules, California, United States) per manufacturer's instructions. Nuclear extracts were prepared by hypotonic lysis (Offterdinger et al. 2002) from 10^10^ HeLa-S3 cells, obtained as frozen cell pellets from the National Cell Culture Center. For each affinity purification, we incubated 50 mg of nuclear lysate proteins with 2 ml of either emerin beads (0.5 mg/ml) or BSA beads in binding buffer (50 mM HEPES, 250 mM NaCl, 0.1% Triton X-100) for 4 h at 4 °C. Beads were collected by centrifugation at 500*g,* washed five times with binding buffer, and eluted with SDS-PAGE sample buffer. Dr. Robert Cole at the Johns Hopkins Mass Spectrometry Facility performed MALDI-TOF. Actin and αII-spectrin were two of seven emerin-associated proteins identified unambiguously in this work, which will be reported separately (J. M. H. and K. L. W., unpublished data).

#### F-actin-binding assays

F-actin columns were assembled as described (Forero and Wasserman 2000). Equal amounts of purified recombinant wild-type and mutant emerin proteins (residues 1–222) were incubated with each column in PBS containing 0.1% Triton X-100 (PBST) for 1 h at 22 °C. After washing beads five times with PBST, bound proteins were eluted and resolved by SDS-PAGE, and detected either by Coomassie blue staining, or by immunoblotting with rabbit serum 2999 against emerin.

Coimmunoprecipitation assays were performed as described (Lee et al. 2001). Briefly, equal masses (5 μg) of actin and either wild-type or mutant emerin were incubated 2 h, then incubated 4 h with protein-A Sepharose-coupled antibodies against emerin or actin. The beads were washed five times with buffer, and bound proteins were eluted with SDS-sample buffer, resolved by SDS-PAGE, then blotted and probed with antibodies specific for either actin or emerin.

To measure emerin binding to single actin filaments, actin was polymerized by the addition of KMEI buffer (2 mM MgCl, 50 mM KCl, 10 mM imidazole [pH 7. 0], and 2 mM EGTA). After 30 min, the indicated form of emerin (4 μM, recombinant wild-type or mutant emerin residues 1–222, with or without conjugation to Alexa-488) was added to the filaments and incubated 30 min; we lastly added Alexa-546 phalloidin (final concentration, 0. 33 μM). These red F-actin polymers were then pelleted at 100,000*g* for 60 min. For experiments with unlabeled emerin, corresponding load, supernatant, and pellet fractions were resolved by SDS-PAGE and stained with Coomassie blue. For experiments with phalloidin-546-labeled F-actin, filaments were viewed using a Zeiss Axiovert 200 fluorescent microscope (Zeiss, Oberkochen, Germany) and images captured using a Quantix CCD camera (Photometrics, Huntington Beach, California, United States) attached to an Apple G4 computer using IPLab (version 3.6; Scanalytics, Fairfax, Virginia, United States) software.

#### Actin polymerization and depolymerization assays

Actin polymerization assays were performed per manufacturer's instructions (Cytoskeleton). Rabbit actin (2 μM; Cytoskeleton, catalog #AKL95) plus pyrene–actin (0.1 μM; Cytoskeleton, catalog #AP05) were used in all assays, unless otherwise stated. Actin polymerization was measured in a fluorimeter (Fluoromax 2; SPEX, Edison, New Jersey, United States), with excitation wavelength 365 nm and emission wavelength 407 nm, and plotted using DataMax-Std (version 2.2; SPEX, Edison, New Jersey, United States). Graphs were refined using Cricketgraph III (version 1.0, Computer Associates, Smithfield, Rhode Island, United States) and Kaleidagraph (version 3.5.1, Synergy Software, Reading, Pennsylvania, United States). Pyrene–actin was always present at 5% of total actin. Increasing concentrations of recombinant emerin were added just prior to initiating actin polymerization. The actin depolymerization assays were performed exactly as described (Mullins et al. 1998) with increasing amounts of recombinant emerin protein. Briefly, F-actin was polymerized by adding 2 μM G-actin to gelsolin–actin seeds (100 nM), then diluted 10-fold in the absence or presence of increasing concentrations of recombinant emerin (residues 1–222). Both gelsolin and actin were obtained from Cytoskeleton (catalog #HPG5 and #AKL95, respectively). Gelsolin–actin seeds were made exactly as described (Blanchoin et al. 2000). Alternatively, 2 μM G-actin was polymerized in the absence of gelsolin for 2 h. Polymerized filaments were then incubated with or without 100 nM CapZ. Subsequent polymerization and depolymerization assays were assayed as described above.

Actin polymerization in the presence or absence of emerin was monitored by fluorescent microscopy, as described (Blanchoin et al. 2000; Amann and Pollard 2001). Samples were diluted 1:500–1:1000, viewed on a Nikon Eclipse E600W microscope (Nikon, Tokyo, Japan), and images were captured with a Q-imaging Retiga Exi CCD camera (Q Imaging, Burnaby, British Columbia, Canada) using IPLab (version 3.9.2) attached to an Apple G5 computer. Images were converted to TIFF images and lengths of filaments were measured in Photoshop version 7.0 (Adobe Systems, San Jose, California, United States).

## Abbreviations

Arp: actin-related protein
BAF: barrier-to-autointegration factor
BSA: bovine serum albumin
EDMD: Emery-Dreifuss muscular dystrophy
F-actin: filamentous actin
GCL: germ cell-less
SR: spectrin-repeat
